# Real-world performance and accuracy of stress echocardiography: the EVAREST observational multi-centre study

**DOI:** 10.1093/ehjci/jeab092

**Published:** 2021-06-20

**Authors:** William Woodward, Cameron Dockerill, Annabelle McCourt, Ross Upton, Jamie O'Driscoll, Katrin Balkhausen, Badrinathan Chandrasekaran, Soroosh Firoozan, Attila Kardos, Kenneth Wong, Gary Woodward, Rizwan Sarwar, Nikant Sabharwal, Elena Benedetto, Nancy Spagou, Rajan Sharma, Daniel Augustine, Apostolos Tsiachristas, Roxy Senior, Paul Leeson, the EVAREST Investigators, EVAREST Investigators, Henry Boardman, Joanna d’Arcy, Abraheem Abraheem, Sanjay Banypersad, Christopher Boos, Sudantha Bulugahapitiya, Jeremy Butts, Duncan Coles, Jacob Easaw, Haytham Hamdan, Shahnaz Jamil-Copley, Gajen Kanaganayagam, Tom Mwambingu, Antonis Pantazis, Alexandros Papachristidis, Ronak Rajani, Muhammad Amer Rasheed, Naveed A Razvi, Sushma Rekhraj, David P Ripley, Kathleen Rose, Michaela Scheuermann-Freestone, Rebecca Schofield, Ayyaz Sultan

**Affiliations:** Cardiovascular Clinical Research Facility, RDM Division of Cardiovascular Medicine, University of Oxford, Oxford OX3 9DU, UK; Cardiovascular Clinical Research Facility, RDM Division of Cardiovascular Medicine, University of Oxford, Oxford OX3 9DU, UK; Cardiovascular Clinical Research Facility, RDM Division of Cardiovascular Medicine, University of Oxford, Oxford OX3 9DU, UK; Cardiovascular Clinical Research Facility, RDM Division of Cardiovascular Medicine, University of Oxford, Oxford OX3 9DU, UK; Ultromics Ltd, Wood Centre for Innovation, OxfordOX3 8SB, UK; Department of Cardiology, St George’s University Hospitals NHS Foundation Trust, London SW17 0QT, UK; School of Human and Life Sciences, Canterbury Christ Church University, Canterbury CT1 1QU, UK; Department of Cardiology, Royal Berkshire Hospitals NHS Foundation Trust, Reading RG1 5AN, UK; Department of Cardiology, Great Western Hospitals NHS Foundation Trust, Swindon SN3 6BB, UK; Department of Cardiology, Buckinghamshire Healthcare NHS Trust, High Wycombe HP11 2TT, UK; Department of Cardiology, Milton Keynes University Hospital NHS Foundation Trust, Milton Keynes MK6 5LD, UK; Lancashire Cardiac Centre, Blackpool Teaching Hospitals NHS Foundation Trust, Blackpool FY3 8NP, UK; Ultromics Ltd, Wood Centre for Innovation, OxfordOX3 8SB, UK; Ultromics Ltd, Wood Centre for Innovation, OxfordOX3 8SB, UK; Oxford Heart Centre, Oxford University Hospitals NHS Foundation Trust, Oxford, OX3 9DU, UK; Oxford Heart Centre, Oxford University Hospitals NHS Foundation Trust, Oxford, OX3 9DU, UK; Cardiovascular Clinical Research Facility, RDM Division of Cardiovascular Medicine, University of Oxford, Oxford OX3 9DU, UK; Ultromics Ltd, Wood Centre for Innovation, OxfordOX3 8SB, UK; Department of Cardiology, St George’s University Hospitals NHS Foundation Trust, London SW17 0QT, UK; Department of Cardiology, Royal United Hospitals NHS Foundation Trust, Bath, BA1 3NG, UK; Health Economic Research Centre, Nuffield Department of Population Health, University of Oxford, Oxford OX3 7LF, UK; National Heart and Lung Institute, Imperial College London, London SW3 6LY, UK; Department of Cardiology, Royal Brompton and Harefield NHS Foundation Trust, London SW3 6NJ, UK; Department of Cardiology, London North West University Healthcare NHS Trust, London HA1 3UJ, UK; Cardiovascular Clinical Research Facility, RDM Division of Cardiovascular Medicine, University of Oxford, Oxford OX3 9DU, UK; Department of Cardiology, Milton Keynes University Hospital NHS Foundation Trust, Milton Keynes MK6 5LD, UK; Oxford Heart Centre, Oxford University Hospitals NHS Foundation Trust, Oxford, OX3 9DU, UK; Oxford Heart Centre, Oxford University Hospitals NHS Foundation Trust, Oxford, OX3 9DU, UK; Department of Cardiology, Tameside and Glossop Integrated Care NHS Foundation Trust, Ashton-under-Lyne, UK; Department of Cardiology, East Lancashire Hospitals NHS Trust, Burnley, UK; Department of Cardiology, Poole Hospital NHS Foundation Trust, Poole, UK; Department of Cardiology, Bradford Teaching Hospitals NHS Foundation Trust, Bradford, UK; Department of Cardiology, Calderdale and Huddersfield NHS Foundation Trust, Calderdale, UK; Department of Cardiology, Mid Essex NHS Hospital Services NHS Trust, Broomfield, UK; Department of Cardiology, Royal United Hospitals NHS Foundation Trust, Bath, BA1 3NG, UK; Department of Cardiology, Wrightington, Wigan and Leigh NHS Foundation Trust, Wigan, UK; Department of Cardiology, Nottingham University Hospitals NHS Trust, Nottingham, UK; Department of Cardiology, Chelsea and Westminster Hospital NHS Foundation Trust, London, UK; Department of Cardiology, The Mid Yorkshire Hospitals NHS Trust, Pinderfields, UK; Department of Cardiology, North Middlesex University Hospital NHS Trust, London, UK; Department of Cardiology, King's College Hospital NHS Foundation Trust, London, UK; Department of Cardiology, Guy’s and St Thomas’ NHS Foundation Trust, London, UK; Department of Cardiology, Yeovil District Hospital NHS Foundation Trust, Yeovil, UK; Department of Cardiology, East Suffolk and North Essex NHS Foundation Trust, Ipswich, UK; Department of Cardiology, Nottingham University Hospitals NHS Trust, Nottingham, UK; Department of Cardiology, Northumbria Healthcare NHS Foundation Trust, North Tyneside, UK; Department of Cardiology, Northampton General Hospital NHS Trust, Northampton, UK; Department of Cardiology, Hampshire Hospitals NHS Foundation Trust, Basingstoke, UK; Department of Cardiology, North West Anglia NHS Foundation Trust, Peterborough, UK; Department of Cardiology, Wrightington, Wigan and Leigh NHS Foundation Trust, Wigan, UK

**Keywords:** Stress echocardiography, Coronary artery disease, Ischaemic heart disease, Accuracy

## Abstract

**Aims:**

Stress echocardiography is widely used to identify obstructive coronary artery disease (CAD). High accuracy is reported in expert hands but is dependent on operator training and image quality. The EVAREST study provides UK-wide data to evaluate real-world performance and accuracy of stress echocardiography.

**Methods and results:**

Participants undergoing stress echocardiography for CAD were recruited from 31 hospitals. Participants were followed up through health records which underwent expert adjudication. Cardiac outcome was defined as anatomically or functionally significant stenosis on angiography, revascularization, medical management of ischaemia, acute coronary syndrome, or cardiac-related death within 6 months. A total of 5131 patients (55% male) participated with a median age of 65 years (interquartile range 57–74). 72.9% of studies used dobutamine and 68.5% were contrast studies. Inducible ischaemia was present in 19.3% of scans. Sensitivity and specificity for prediction of a cardiac outcome were 95.4% and 96.0%, respectively, with an accuracy of 95.9%. Sub-group analysis revealed high levels of predictive accuracy across a wide range of patient and protocol sub-groups, with the presence of a resting regional wall motion abnormalitiy significantly reducing the performance of both dobutamine (*P* < 0.01) and exercise (*P* < 0.05) stress echocardiography. Overall accuracy remained consistently high across all participating hospitals.

**Conclusion:**

Stress echocardiography has high accuracy across UK-based hospitals and thus indicates stress echocardiography is being delivered effectively in real-world practice, reinforcing its role as a first-line investigation in the assessment of patients with stable chest pain.

## Graphical Abstract

**Figure:**
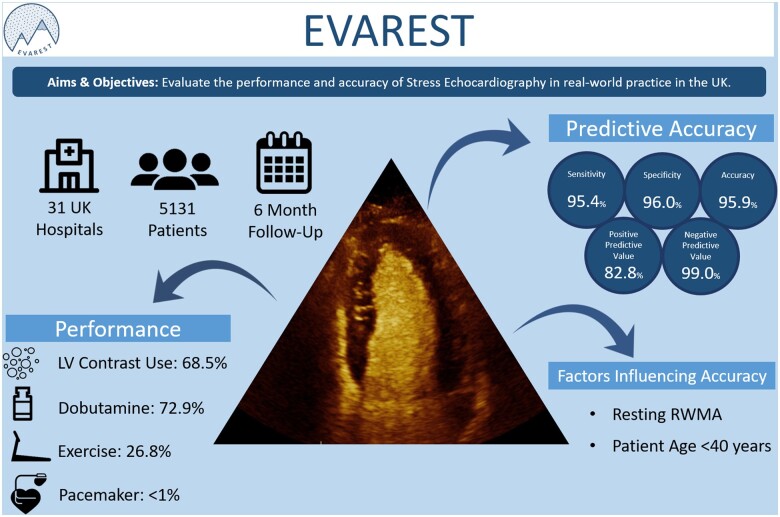

## Introduction

Functional imaging has equal prominence with non-invasive anatomical imaging for the diagnosis of coronary artery disease (CAD) in guidance issued by the European Society of Cardiology.^1^ In the UK, NICE proposes non-invasive anatomical imaging for first-line investigation with functional imaging for second-line investigation.^2^ However, lack of infrastructure and trained personnel^3^ means in real-world practice functional imaging remains the main first-line test for CAD.^4^^,^^5^

Global reliance on functional imaging as the first-line investigation for CAD, particularly when this approach differs from some national guidelines, raises concerns about whether current patient care is optimal. However, evaluation of individual imaging tests in guidelines has tended to need to rely on meta-analysis of small experimental studies^6–8^ or, in the case of stress echocardiography, historical studies from the 1990s and 2000s.^6^^,^^9^

Recent large-scale randomized clinical trials, such as PROMISE, show similar outcomes with either an anatomical or functional imaging approach,^10^ and contemporary single centre observational studies indicate good performance of stress echocardiography for diagnosis and prognostication.^11^^,^^12^ Furthermore recent studies such as ISCHEMIA,^13^ combined with evidence from COURAGE,^14^ demonstrate the non-inferiority of a medical therapy-first strategy compared with an initial invasive strategy. Whilst other large-scale, prospective studies have examined the accuracy of stress echocardiography in other regions across the world,^15–20^ the EVAREST (Echocardiography: Value and Accuracy at Rest and Stress) study is the first such large-scale evaluation of the use and accuracy of stress echocardiography in clinical practice within the National Health Service in the UK. The participating centres are representative of the geographical variation, hospital size, and patient demographics seen within the UK. In this real-world practice, we describe stress echocardiogram protocol performance, as well as accuracy and patient outcome based on all those with 6-month outcome data by January 2021.

## Methods

### Study design

EVAREST is a prospective, multi-centre, observational study. Following a pilot project in Oxford (OxCardioFuse, IRAS reference: 08/H0604/127), recruitment commenced in the main study (ClinicalTrials.gov ID: NCT03674255). Details about hospital recruitment are provided in Supplementary data online.

### Participants

Patients undergoing stress echocardiography for evaluation of stable chest pain were recruited from 28 NHS Trusts, comprising 31 hospitals, between March 2015 and March 2020. All patients provided informed consent. Ethical approval was granted by the Health Research Authority NRES Committee (South Central—Berkshire) review board (IRAS reference: 14/SC/1437). This study was conducted in accordance with the Declaration of Helsinki.

### Procedures

Tests were conducted and reported in accordance with each hospital’s standard protocol; mode of stress and contrast use were at the operator’s discretion. Procedure details, results, and participant medical history were obtained from medical records and recorded on an electronic database (Castor EDC, Amsterdam, Netherlands).

### Outcomes

Follow-up is ongoing with a proportion of patients consented to follow-up for 10 years. Initial follow-up included a medical record review and patient telephone call completed towards the end of the first year after recruitment. Cardiac imaging, procedure reports, or death certification were obtained, if applicable, and data extracted including the location of coronary disease, if available. All angiogram reports were reviewed and diameter stenosis (as visually assessed by the operator) was recorded for each coronary artery. Analysis presented in this article is based on the full dataset with follow-up censored at 6 months.

All clinical data were reviewed by an adjudication committee, including at least one accredited cardiologist, blinded to stress echocardiogram results and a binary (cardiac/non-cardiac) outcome assigned (Supplementary data online, *Figure S1*). Cardiac outcome was defined as angiography demonstrating an anatomically or functionally significant lesion [defined as greater than 70% narrowing (or 50% in the left main stem) or abnormal fractional flow reserve or instantaneous wave-free ratio], referral for revascularization, initiation of appropriate pharmacological therapy, acute coronary syndrome, or cardiac-related death. All patients in whom no additional cardiac intervention, management, or investigation was required were assigned a non-cardiac outcome.

When assessing the accuracy of stress echo to identify the location of the coronary disease, each segment was assigned a supplying artery (as per Elhendy *et al.*^21^). The basal, mid and apical anterior wall, basal and mid-anteroseptum, mid-inferoseptum, apical septum, apical lateral, and apical cap, were assigned to the left anterior descending (LAD) coronary artery territory. The basal and mid-lateral, and basal and mid-inferolateral walls were assigned to the left circumflex artery (LCx) territory. The basal, mid and apical inferior, and basal inferoseptum were assigned to the right coronary artery (RCA) territory.

### Statistical analysis

Patient demographics and stress echocardiogram protocols were reported using standard approaches. Descriptive statistics were investigated as frequencies and medians [interquartile range (IQR)]. Normality was assessed by Shapiro–Wilk test. Sub-group comparisons were made by Mann–Whitney or χ^2^ tests, as appropriate. Association of patient demographics or test protocol on contrast usage and accuracy of stress echocardiography were tested with odds ratios (OR) and 95% confidence intervals (CIs) in multivariate logistic regressions. Kaplan–Meier survival curves and Log-Rank tests were used to study differences in cardiac outcomes between groups. A Cox proportional hazard model was used to estimate the hazard ratio (HR) of a positive stress echocardiogram and ischaemic burden, after adjusting for cardiac risk factors and resting regional wall motion abnormalities. To compare outcomes against stress echocardiogram results the stress echocardiogram was defined as either true positive, true negative, false positive, or false negative. Sensitivity, specificity, positive predictive value, and negative predictive value were calculated using standard approaches for stress echocardiography overall, and for sub-groups based on patient characteristics and stress echocardiogram protocol (provided the sub-group contained at least 50 patients). Receiver operating characteristic (ROC) curves were plotted for each sub-group, and the area under the ROC curve (AUROC) was calculated. AUROCs were compared by a χ^2^ test to determine differences in predictive accuracy between patient and protocol sub-groups. Univariate logistic regression, Kruskal–Wallis and Mann–Whitney tests were used to investigate coronary vessel-specific accuracy. All statistical analysis was carried out using STATA IC 15 (STATA Corp. LLC, TX, USA).

## Results

**Figure 1:**
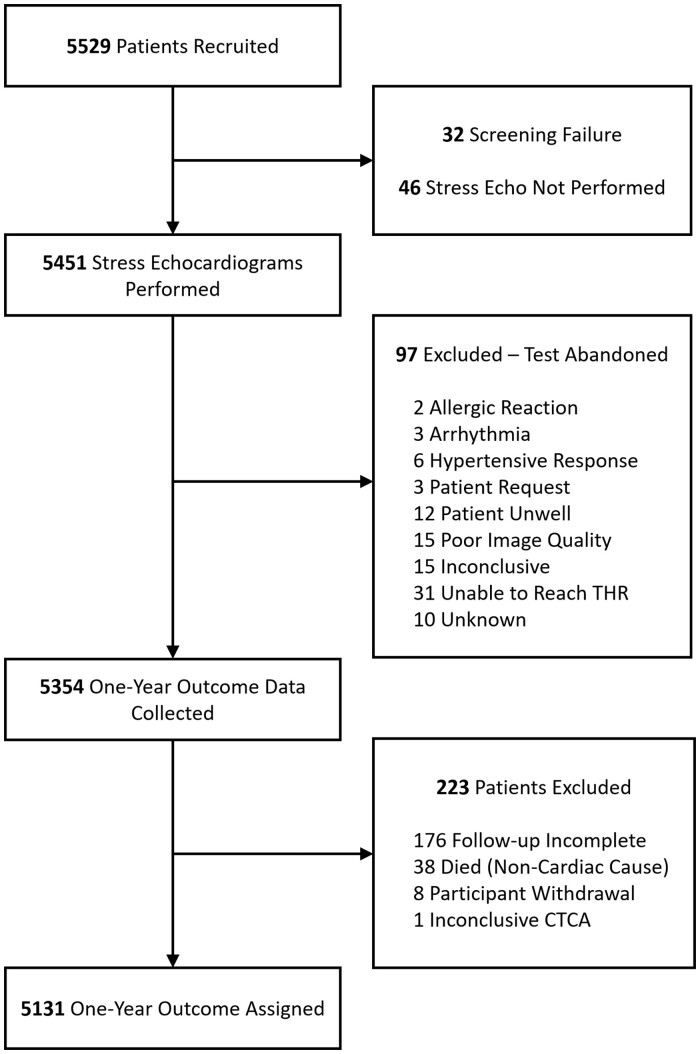
Recruitment flow chart of the first 5529 patients recruited into the OxCardioFuse and EVAREST studies. THR, target heart rate; CTCA, computed tomography coronary angiogram.

*Figure 1* shows patient recruitment from 31 hospitals. The broad geographical distribution of this research network and hospital characteristics are shown in the Supplementary data online (Supplementary data online, *Figure S2*). Of those recruited, 32 were identified as screening failures and 46 were excluded from the analysis as their stress echocardiogram was not performed. A further 97 patients were excluded as their stress echocardiogram was inconclusive or abandoned. Of the 5354 patients who were followed up, 223 were excluded. Therefore, a total of 5131 patients were included in the analysis.

Patient demographics are reported in *Table 1*. Median age was 65 years (IQR 57–74) and 2823 (55%) were male. Stress echocardiograms were negative for inducible ischaemia in 4139 (80.7%) patients and positive in 992 (19.3%). *Table 1* shows that patients with a positive test were more likely to have cardiac risk factors including male sex, increased age, increased body mass index (BMI), hypertension, hypercholesterolaemia, diabetes mellitus, and pre-existing vascular disease. Pre-existing CAD was present in 1868 (36.7%) participants, with 867 (17.2%) participants having previously suffered a myocardial infarction.

**Table 1:** Patient demographics at time of stress echocardiogram

	Positive stress echo (*n* = 992)	Negative stress echo (*n* = 4139)	***P*-value** ^*^	**Overall cohort (*n* = 5131)**
Male (%)	636 (64.1)	2187 (52.8)	<0.0001	2823 (55.0)
Median age (years) (IQR)	68 (59–74)	66 (56–73)	<0.0001	65 (57–74)
Median BMI (kg/m^2^) (IQR)	28.7 (25.7–32.1)	28.2 (25.0–31.7)	<0.05	28.3 (25.1–31.8)
Smoking				
Current smoker (%)	143/965 (14.8)	501/3987 (12.6)	0.062	644/4952 (13.0)
Ex-smoker (%)	355/965 (36.8)	1428/3987 (35.8)	0.573	1783/4952 (36.0)
Hypertension (%)	473/970 (48.8)	1724/3977 (43.3)	<0.01	2197/4947 (44.4)
Hypercholesterolaemia (%)	456/970 (47.0)	1385/3977 (34.8)	<0.0001	1841/4947 (37.2)
Diabetes mellitus (%)	199/970 (20.5)	654/3977 (16.4)	<0.01	853/4947 (17.2)
Family history of premature CAD (%)	5/970 (0.5)	67/3977 (1.7)	<0.01	72/4947 (1.5)
Peripheral vascular disease (%)	44/970 (4.5)	113/3977 (2.8)	<0.001	157/4947 (3.2)
Pre-existing CAD (%)	500/984 (50.8)	1368/4104 (33.3)	<0.0001	1868/5088 (36.7)
Previous MI (%)	260/976 (26.6)	607/4071 (14.9)	<0.0001	867/5047 (17.2)
Previous PCI (%)	410/980 (41.8)	1137/4076 (27.9)	<0.0001	1547/5056 (30.6)
Previous CABG (%)	147/980 (15.0)	240/4084 (5.9)	<0.0001	387/5046 (7.6)
Current medications				
ACEi/ARB (%)	212/989 (21.4)	678/4132 (16.4)	<0.0001	890/5121 (17.4)
Aspirin (%)	277/989 (28.0)	784/4132 (19.0)	<0.0001	1061/5121 (20.7)
Beta-blocker (%)	194/989 (19.6)	654/4132 (15.8)	<0.01	848/5121 (16.6)
Calcium channel blocker (%)	125/989 (12.6)	496/4132 (12.0)	0.583	621/5121 (12.1)
Nitrates (%)	208/989 (21.0)	719/4132 (17.4)	<0.01	927/5121 (18.1)
Statin (%)	455/989 (46.0)	1616/4132 (39.1)	<0.0001	2071/5121 (40.4)
Resting RWMA (%)	304/990 (30.7)	402/4131 (9.7)	<0.0001	706/5121 (13.8)

Patient demographics at time of stress echocardiogram for 5131 patients with outcome data. Presented as n./total n. Percentages quoted in brackets.

* *P*-value for comparison of demographics between positive and negative stress echocardiography.BMI, body mass index; CAD, coronary artery disease ; MI, myocardial infarction; PCI, percutaneous coronary intervention; CABG, coronary artery bypass graft surgery; ACEi/ARB, Angiotensin converting enzyme inhibitor/angiotensin receptor blocker; RWMA, regional wall motion abnormalities.

Dobutamine was the most common stressor accounting for 3739 (72.9%) of tests, while exercise was used in 1375 (26.8%) studies. Of those undergoing exercise stress echocardiography, 918 (66.8%) underwent treadmill stress, whilst 454 (33.0%) underwent bicycle ergometer stress, mode of stress was not recorded for 3 (0.2%) patients. Seventeen patients (0.2%) underwent a pacemaker-mediated study. Supplementary data online, *Table S1* shows a higher prevalence of cardiovascular risk factors in those undergoing dobutamine stress echocardiograms, compared to those having exercise studies. Left ventricular (LV) contrast was used in 3510 (68.5%) of studies, with more frequent use in dobutamine stress echocardiograms compared to exercise stress (76.1% vs. 47.8%). Increased age and BMI were independently associated with contrast use in multivariate regression analysis (Supplementary data online, *Table S2*).

**Figure 2:**
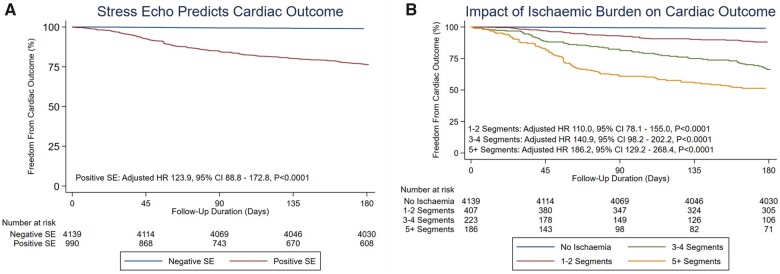
Kaplan–Meier plots demonstrating freedom from cardiac outcome. (*A*) Plot separated by stress echocardiogram result. (*B*) Plot separated by burden of ischaemia, with an increased adjusted hazard ratio associated with a larger burden of ischaemia.

Six-month outcome data were analysed to determine the predictive accuracy of stress echocardiography. *Figure 2A* demonstrates time-related events up to 6 months after stress echocardiography. A positive stress echocardiogram was significantly associated with cardiac outcome (adjusted HR 123.9, 95% CI 88.8–172.8; *P* < 0.0001).

Overall sensitivity for all types of stress echocardiography and patent was 95.4% with a specificity of 96.0%. Positive predictive value and negative predictive value were 82.8% and 99.0%, respectively. Overall accuracy was 95.9%. No significant difference in predictive ability was observed between dobutamine and exercise stress echocardiography (*P* = 0.533). *Table 2* shows the sensitivity, specificity, and accuracy for stress echocardiography when separated by sub-group according to patient characteristic and type of stressor. The presence of a resting regional wall motion abnormality was associated with a significant reduction in overall predictive accuracy in both exercise stress echocardiography (*P* < 0.05) and dobutamine stress echocardiography (*P* < 0.01). The presence of left bundle branch block (LBBB), which is more common in those with resting wall motion abnormalities, also reduced sensitivity, specificity, and accuracy during dobutamine stress echocardiography. However, there was no statistically significant difference in predictive performance (*P* = 0.366). The presence of atrial fibrillation selectively reduced sensitivity of dobutamine stress echocardiography but overall predictive ability did not change (*P* = 0.728). Sensitivity was higher for both dobutamine and exercise stress echocardiography in those with the previous coronary artery bypass graft surgery but specificity was lower resulting in no overall change in predictive ability for either dobutamine (*P* = 0.813) or exercise stress echocardiography (*P* = 0.982). Increased BMI > 40 kg/m^2^ did not significantly impact overall performance (*P* = 0.402); however, sensitivity was higher during dobutamine stress echocardiography in those patients with a BMI of <40 kg/m^2^. Overall predictive ability was significantly greater (*P* < 0.0001) in patients aged <40 years. Sub-group analysis was carried out on patients undergoing stress echocardiography prior to surgery. No significant differences (*P* = 0.562) in predictive accuracy were observed in this group of patients.

**Table 2:** Diagnostic performance of dobutamine and exercise stress echocardiography

	N		Sensitivity (%)		Specificity (%)		Accuracy (%)		*P*-value	
	DSE	ESE	DSE	ESE	DSE	ESE	DSE	ESE	DSE	ESE
Overall	3739	1375	95.6	94.4	96.0	96.0	96.0	95.8	**·**	**·**
Normal resting wall motion	3168	1238	96.4	94.0	96.6	96.9	96.6	96.5	0.002	0.048
Resting RWMA	564	134	94.2	95.9	91.5	84.7	92.6	88.8		
Normal conduction	3641	1347	95.7	94.8	96.1	96.0	96.0	95.8	0.366	**·**
LBBB	60	17	90.9	**·**	91.8	**·**	91.7	**·**		
RBBB	38	11	**·**	**·**	**·**	**·**	**·**	**·**	**·**	
Sinus rhythm	2314	808	93.9	92.9	96.5	97.3	96.1	96.7	0.728	**·**
Atrial fibrillation	131	14	90.9	**·**	97.2	**·**	96.2			
No previous CABG	3400	1307	95.5	93.6	96.1	96.2	96.0	95.9	0.813	0.982
Previous CABG	316	67	96.2	100.0	94.8	89.7	95.3	94.0		
BMI < 40 kg/m^2^	3246	1307	96.1	94.7	96.0	96.2	96.1	95.9	0.402	**·**
BMI > 40 kg/m^2^	182	30	92.9	**·**	94.8	**·**	94.5	**·**		
Age < 40 years	74	61	100.0	100.0	98.6	98.3	98.6	98.4	0.000	0.001
Age > 40 years	3651	1313	95.7	94.4	96.0	95.9	95.9	95.7		
Indication: ischaemia	3642	1360	95.7	94.4	96.1	96.1	96.0	95.8	0.562	**·**
Indication: pre-operative/pre-transplant	97	15	94.4	**·**	93.7	**·**	93.8	**·**		

Diagnostic performance of stress echocardiography, overall and by patient sub-group. Values are presented for dobutamine stress echocardiography (DSE) and exercise stress echocardiography (ESE). *P*-values for χ^2^ comparison of AUROCs between sub-groups. NB. **·** indicates that fewer than 50 patients were in this sub-group, therefore values not calculated.

BMI, body mass index; CABG, coronary artery bypass graft surgery; LBBB, left bundle branch block; RBBB, right bundle branch block; RWMA, regional wall motion abnormalities.

*Table 3* reports the accuracy of stress echocardiography related to contrast use. No statistically significant differences in overall accuracy were observed between contrast and non-contrast stress echocardiograms (*P* = 0.813). A significant (*P* < 0.05) reduction in predictive accuracy was observed with non-contrast exercise stress echocardiography in patients with abnormal resting wall motion, compared with contrast-enhanced stress echocardiography. This related to a higher specificity when contrast was used. However, no difference was observed between contrast and non-contrast stress echocardiography when resting wall motion was normal (*P* = 0.616).

**Table 3:** Diagnostic performance of contrast and non-contrast stress echocardiography

	*N*		Sensitivity (%)		Specificity (%)		Accuracy (%)		*P*-value	
	DSE	ESE	DSE	ESE	DSE	ESE	DSE	ESE	DSE	ESE
Contrast stress echocardiography										
Overall	2841	656	95.6	94.5	96.1	95.9	96.0	95.7	**·**	**·**
Normal resting wall motion	2396	596	96.4	93.8	96.6	97.6	96.6	97.2	0.010	0.006
Resting RWMA	439	59	94.3	96.3	91.7	68.8	92.7	81.4		
Normal conduction	2763	648	95.7	94.4	96.2	96.1	96.1	95.8	**·**	**·**
LBBB	44	6	**·**	**·**	**·**	**·**	**·**	**·**		
RBBB	34	2	**·**	**·**	**·**	**·**	**·**	**·**		
Sinus rhythm	2066	556	95.2	93.3	96.3	97.0	96.1	96.5	0.900	**·**
Atrial fibrillation	117	7	95.2	**·**	96.9	**·**	96.6	**·**		
No previous CABG	2595	625	95.6	93.7	96.2	96.2	96.1	95.8	0.561	**·**
Previous CABG	227	31	95.4	**·**	94.4	**·**	94.7	**·**		
BMI < 40 kg/m^2^	2447	614	95.9	94.3	96.2	96.4	96.2	96.1	0.733	**·**
BMI > 40 kg/m^2^	155	22	95.8	**·**	94.7	**·**	94.8	**·**		
Age < 40 years	62	25	100.0	**·**	98.3	**·**	98.4	**·**	0.001	**·**
Age > 40 years	2765	630	95.8	94.4	96.0	95.9	96.0	95.7		
Indication: ischaemia	2755	648	95.6	94.4	96.2	96.1	96.1	95.8	0.468	**·**
Indication: pre-operative/pre-transplant	86	8	94.4	**·**	92.7	**·**	93.0	**·**		
Non-contrast stress echocardiography										
Overall	892	716	95.6	94.4	95.9	96.1	95.9	95.8	**·**	**·**
Normal resting wall motion	767	640	96.3	94.2	96.5	96.3	96.5	95.9	0.110	0.914
Resting RWMA	124	75	93.6	95.5	90.9	94.3	91.9	94.7		
Normal conduction	872	696	95.6	95.0	95.8	96.0	95.8	95.8	**·**	**·**
LBBB	16	11	**·**	**·**	**·**	**·**	**·**	**·**		
RBBB	4	9	**·**	**·**	**·**	**·**	**·**	**·**		
Sinus rhythm	246	239	79.3	91.9	98.6	98.0	96.3	97.1	**·**	**·**
Atrial fibrillation	14	7	**·**	**·**	**·**	**·**	**·**	**·**		
No previous CABG	799	679	95.1	93.6	95.9	96.3	95.7	95.9	0.570	
Previous CABG	89	36	97.6	**·**	95.8	**·**	96.6	**·**		
BMI < 40 kg/m^2^	795	692	96.5	95.1	95.5	96.0	95.7	95.8	**·**	**·**
BMI > 40 kg/m^2^	27	8	**·**	**·**	**·**	**·**	**·**	**·**		
Age < 40 years	12	36	**·**	**·**	**·**	**·**	**·**	**·**	**·**	**·**
Age > 40 years	880	680	95.6	94.4	95.8	95.9	95.8	95.6		
Indication: ischaemia	881	709	95.6	94.3	95.9	96.1	95.8	95.8	**·**	**·**
Indication: pre-operative/pre-transplant	11	7	**·**	**·**	**·**	**·**	**·**	**·**		

Diagnostic performance of stress echocardiography, overall and by patient sub-group, separated by contrast and non-contrast scans. Values are presented for dobutamine stress echocardiography (DSE) and exercise stress echocardiography (ESE). *P*-values for χ^2^ comparison of AUROCs between sub-groups. NB. **·** indicates that fewer than 50 patients were in this sub-group, therefore values not calculated.

BMI, body mass index; CABG, coronary artery bypass graft surgery; LBBB, left bundle branch block; RBBB, right bundle branch block; RWMA, regional wall motion abnormalities.

In the 21 hospitals that recruited more than 50 patients, the diagnostic performance of stress echocardiography was determined by calculating AUROCs. These ranged from 0.900 to 1.000, with a mean AUROC of 0.9494, demonstrating that stress echocardiography is being performed to a high diagnostic standard at all centres. Comparison of the AUROCs between centres, however, did reveal a statistically significant difference in accuracy (*P* < 0.0001).

**Figure 3:**
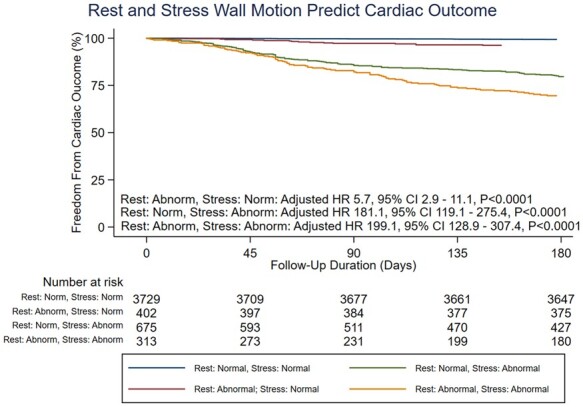
Kaplan–Meier curves demonstrating that hazard of cardiac outcome increases with the presence of inducible ischaemia. However, the presence of resting regional wall motion abnormalities conveys an increased hazard of cardiac outcome, both with and without the presence of inducible ischaemia on stress echocardiography.

The presence of a resting regional wall motion abnormality was significantly associated with the likelihood of having a positive stress echocardiogram, with an adjusted odds ratio of 4.1 (95% CI 3.5–4.9) (*P* < 0.0001). Of those stress echocardiograms that were positive for inducible ischaemia, 30.7% had resting wall motion abnormalities, compared with 9.7% of negative stress echocardiograms. The presence of a resting regional wall motion abnormality was also significantly associated with the likelihood of severe coronary disease on angiography, with an adjusted HR of 2.8 (95% CI 2.4–3.3) (*P* < 0.0001). *Figure 3* demonstrates how the occurrence of severe coronary disease differs based on both the presence of resting regional wall motion abnormalities and the presence of inducible ischaemia.

The median number of ischaemic segments identified during a positive stress echocardiogram was 3 (IQR 2–4) (see Supplementary data online, *Figure S3*). *Figure 2B* demonstrates a significant separation in outcomes over 6 months according to number of ischaemic segments (*P* < 0.0001). Those patients with a positive stress echocardiogram who were managed medically but subsequently presented with acute coronary syndromes had the significantly higher ischaemic burden at baseline compared to those who were managed medically with no further cardiac events [four segments (IQR 3–6) vs. two segments (IQR 1–3), *P* < 0.01]. Ischaemic burden was significantly higher in patients referred for angiography compared to those managed medically [four segments (IQR 2–5) and two segments (IQR 1–3), *P* < 0.0001] and those found to have angiographically severe disease had a higher ischaemic burden compared to those with non-obstructive disease [four segments (IQR 3–6) and three segments (IQR 2–4), *P* < 0.0001). Single-vessel disease was present in 114 patients with positive stress echocardiograms, whilst 93 patients had multi-vessel disease. Details of location are provided in the Supplementary data online, *Results*. Overall ischaemic burden was greater with multi-vessel disease compared to single-vessel disease (*P* < 0.05), five segments (IQR 3–7 segments) compared with four segments (IQR 2–6 segments), respectively. No significant difference (*P* = 0.118) in ischaemic burden was observed between LAD, LCx, and RCA disease. Univariate logistic regression demonstrated that LAD ischaemia was significantly associated with ischaemia in the LAD territory (OR 4.9, 95% CI 1.9–13.0; *P* < 0.001) whilst RCA ischaemia was significantly associated with RCA territory ischaemia (OR 2.4, 95% CI 1.3–4.3; *P* < 0.01). However, stress echocardiography lacked the precision to detect LCx disease, with no significant association with LCx ischaemia (OR 1.5, 95% CI 0.8–2.8; *P* = 0.156).

## Discussion

This study provides contemporary, real-world data on the use, and accuracy of stress echocardiography in clinical practice across a national healthcare system. When used as the first-line test for the evaluation of CAD, outcomes for patients are consistent, or better, than reported as best practice from randomized controlled trials of anatomical^22–24^ or functional imaging.^8^^,^^10^ Across hospitals of varying sizes, activity levels, and locations, stress echocardiography was performed consistently to a high standard. It is noteworthy that only 1.8% of stress echocardiograms were considered non-diagnostic.

Historically, significant variability in the performance of stress echocardiography has been reported between different studies with sensitivity and specificity ranging from 33 to 96% and 38 to 97%, respectively.^2^^,^^6^^,^^25^ National echocardiography societies have therefore prioritized education, training, and monitoring of competence.^20^^,^^26^ These initiatives could explain why this study shows delivery of stress echocardiography to a high standard with high levels of clinically meaningful sensitivity and specificity within the UK. Protocol selection may also partly be responsible. Dobutamine stress echocardiography was more commonly used than exercise stress and, although operator experience with exercise and local facilities may drive this difference,^4^ use of dobutamine was associated with the presence of a higher BMI, increased age, and a greater number of cardiac risk factors, suggesting a degree of stressor selection to optimize the procedure.

Benefits of stress echocardiography include a lack of ionizing radiation, which complicates other cardiac imaging modalities. However, image quality can be adversely affected by patient body habitus, making interpretation challenging. One study reports up to one in three patients may have sub-optimal images.^27^ This can be overcome with LV contrast agents.^28^ We observed a high use of LV contrast, at 68.5% of studies; known to increase diagnostic accuracy.^28^ Patients receiving contrast tended to have an elevated BMI and older age, matching known factors likely to increase the requirement for contrast use.^28^ Our findings demonstrate contrast-enhanced stress echocardiography has a high predictive accuracy, even in the sub-group of patients with a BMI >40 kg/m^2^.

Accuracy was mainly affected by non-procedural factors, specifically, pre-existing regional wall motion abnormalities, which are recognized as complicating identification of new wall motion abnormalities^29^ as well as resulting in a higher risk of adverse events.^21^^,^^30^ The reduction in accuracy in those with regional wall motion abnormalities may reflect an impact of dobutamine on post-systolic shortening,^31^ which could disguise a lack of segmental contractile function, leading to misdiagnosis on visual assessment.

We have demonstrated the ability of stress echocardiography to accurately detect flow-limiting coronary disease in the LAD and RCA; however, no significant association was observed between ischaemia detected the LCx territory and LCx coronary disease. This lack of association between LCx ischaemia and corresponding disease on angiography may be explained by the termination of the stress echocardiogram following the development of ischaemia in a different territory with a lower coronary flow reserve. Once ischaemia has been documented, especially in dobutamine stress echocardiography, the test is typically terminated and may therefore mask an ischaemic response in another territory with significant stenosis.

Since stress echocardiography relies on the qualitative assessment of wall motion, accurate interpretation is dependent on operator experience.^32^ One obstacle to a more widespread use of stress echocardiography may be lack of trained operators to confidently and accurately interpret the test. In the future, this obstacle may be overcome by the incorporation of artificial intelligence (AI) tools into the clinic capable of performing a quantitative assessment of stress images.^33–35^ Increased consistency and confidence in reporting by the use of AI could broaden the range of personnel who could perform stress echocardiograms.

Acute coronary events or cardiac-related deaths that occur after a negative stress echocardiogram remain a concern. However, this study shows similar rates of 1–2% of patients having acute events over 6 months in both the negative and positive stress echocardiogram cohorts. Recent trials have shown an early invasive strategy has a similar impact on longer-term event rates as a medical management-based approach,^13^^,^^24^ which may reflect the evolving nature of the underlying pathology and emergence of new disease. As CAD progresses over time, accuracy for stress echocardiography to predict longer-term outcomes is likely to vary and subsequent analysis with longer-term follow-up will be of interest.

The present study reveals over half of patients who have positive functional imaging do not go on to have further investigation or intervention. The number of ischaemic segments was lower in this group consistent with accepted clinical decision making to manage medically those with lower ischaemic burden.^14^ This study confirms a striking graded association between the degree of ischaemia assessed by the clinician and the likelihood of cardiac outcome over the next 6 months. Reassuringly, outcome at 6 months in the medically managed positive stress echocardiogram population was comparable to other arms of clinical care. The recently published ISCHEMIA study would support the medical management of stable ischaemic heart disease patients with preserved ventricular function and no evidence of heart failure or LMS disease, even if they have a large burden of ischaemia.^13^ Long-term follow-up of this study will investigate whether revascularization reduces the incidence of myocardial infarction in the longer term in patients with significant ischaemia.

The study has limitations. Firstly, by using real-world data, angiographic confirmation of obstructive or non-obstructive coronary disease was not available for all patients. Instead, patients were allocated to outcome based on clinical history during a 6-month period, using criteria developed for handling outcomes in this setting.^36^^,^^37^ Therefore, patients with obstructive coronary disease who had a negative stress echocardiogram but then remained well for the next 6 months could have been misclassified from an anatomical perspective in analysis. Arguably, this outcome was clinically acceptable and the statistical misclassification bias is minimized by related misclassification in patients with positive stress echocardiogram who did not undergo further investigation. Secondly, patients who underwent angiography were judged based on the degree of stenosis in their epicardial arteries assessed by the operating clinician rather than an independent review of the angiogram. Thirdly, this meant potential causes of non-obstructive ischaemia, such as microvascular disease, may have been misclassified in outcome allocation as a false positive stress echocardiogram. Fourthly, not all sites started recruiting at the same time and therefore some sites contributed more proportionally to the dataset. Reanalysis at future time points beyond 6 months and with more patients from each site providing outcome data will be of interest. Finally, due to the nature of the consent process, there may be a selection bias amongst the study population compared with other studies using registry or audit data.

In conclusion, the EVAREST study provides a unique insight into the current use and accuracy of stress echocardiography in real-world practice across 31 UK-based hospitals. Stress echocardiography has a consistent and high accuracy across a broad range of hospitals with high diagnostic results, this is reassuring that stress echocardiography is being delivered effectively in real-world practice. The results give confidence that stress echocardiography can safely be used as a first-line investigation in the management of patients presenting with stable chest pain.

## Funding

This work was supported by National Institute for Health Research Health Education England Healthcare Science Research Fellowship [NIHR-HCS-P13-04-001]; Cardiovascular Clinical Research Facility, University of Oxford; Ultromics Ltd.; Lantheus Medical Imaging Inc. and National Institute for Health Research Oxford Biomedical Research Centre, University of Oxford. The funders of the study had no role in study design, data collection, analysis, interpretation, or writing of the report.

**Conflict of interest:** R.U. and G.W. are employees and shareholders of Ultromics which develops AI echocardiography software. P.L. is a shareholder and non-executive director of Ultromics, has previously consulted for Intelligent Ultrasound and has held research grants from the Lantheus Medical Imaging and the NIHR. R.U. and P.L. are inventors on patents in the field of echocardiography. K.B. and R.Sa have received personal fees from Ultromics Ltd. R.Se has received speaker fees from Philips Eindhoven, Holland; Lantheus Medical Imaging, Boston, USA; Bracco, Milan, Italy. All other authors have nothing to declare.

## Data availability

The data underlying this article will be shared on reasonable request to the corresponding author.

## Supplementary Material

jeab092_supplementary_data
