# Supplementary material for: Real-world performance and accuracy of stress echocardiography: the EVAREST observational multi-centre study
Source: Eur Heart J Cardiovasc Imaging. 2021 Jun 20;23(5):689–98. doi: 10.1093/ehjci/jeab092 (PMC9016358; doi:10.1093/ehjci/jeab092)
Supplement: jeab092_supplementary_data [file jeab092_supplementary_data.docx]

**REAL-WORLD PERFORMANCE AND ACCURACY OF STRESS ECHOCARDIOGRAPHY: THE EVAREST OBSERVATIONAL MULTI-CENTRE STUDY**

**SUPPLEMENTAL MATERIAL**

**EVAREST Investigator Group**

Abraheem Abraheem^16^, Daniel Augustine^11^, Katrin Balkhausen^5^, Sanjay Banypersad^17^, Elena Benedetto^1^, Henry Boardman^8, 10^, Christopher Boos^18^, Sudantha Bulugahapitiya^19^, Jeremy Butts^20^, Badrinathan Chandrasekaran^6^, Duncan Coles^21^, Joanna d’Arcy^10^, Cameron Dockerill^1^, Jacob Easaw^11^, Soroosh Firoozan^7^, Haytham Hamdan^22^, Shahnaz Jamil-Copley^23^, Gajen Kanaganayagam^24^, Attila Kardos^8^, Annabelle McCourt^1^, Tom Mwambingu^25^, Jamie O'Driscoll^3,4^, Antonis Pantazis^26^, Alexandros Papachristidis^27^, Ronak Rajani^28^, Muhammad Amer Rasheed^29^, Naveed A. Razvi^30^, Sushma Rekhraj^23^, David P. Ripley^31^, Kathleen Rose^32^, Nikant Sabharwal^10^, Rizwan Sarwar^2,10^, Michaela Scheuermann-Freestone^33^, Rebecca Schofield^34^, Roxy Senior^13,14,15^, Rajan Sharma^3^, Nancy Spagou^2^, Ayyaz Sultan^22^, Apostolos Tsiachristas^12^, Ross Upton^1,2^, Kenneth Wong^9^, Gary Woodward^2^, William Woodward^1^, Paul Leeson^1^.

^1^Cardiovascular Clinical Research Facility, RDM Division of Cardiovascular Medicine, University of Oxford, Oxford, UK, ^2^ Ultromics Ltd, Wood Centre for Innovation, Oxford, UK. ^3^St George’s University Hospitals NHS Foundation Trust, London, UK. ^4^School of Human and Life Sciences, Canterbury Christ Church University, Canterbury, UK. ^5^Royal Berkshire Hospitals NHS Foundation Trust, Reading, UK. ^6^Great Western Hospitals NHS Foundation Trust, Swindon, UK. ^7^Buckinghamshire Healthcare NHS Trust, High Wycombe, UK. ^8^Milton Keynes University Hospital NHS Foundation Trust, Milton Keynes, UK. ^9^Blackpool Teaching

Hospitals NHS Foundation Trust, Blackpool, UK. ^10^Oxford University Hospitals NHS Foundation Trust, Oxford, UK, ^11^Royal United Hospitals NHS Foundation Trust, Bath, UK, ^12^Health Economic Research Centre, Nuffield Department of Population Health, University of Oxford, Oxford, UK, ^13^National Heart and Lung Institute, Imperial College London, UK, ^14^Royal Brompton and Harefield NHS Foundation Trust, London, UK ^15^ London North West University Healthcare NHS Trust, London, UK. ^16^Tameside and Glossop Integrated Care NHS Foundation Trust, Ashton-under-Lyne, UK. ^17^East Lancashire Hospitals NHS Trust, Burnley, UK. ^18^Poole Hospital NHS Foundation Trust, Poole, UK. ^19^Bradford Teaching Hospitals NHS Foundation Trust, Bradford, UK. ^20^Calderdale and Huddersfield NHS Foundation Trust, Calderdale, UK. ^21^Mid Essex NHS Hospital Services NHS Trust, Broomfield, UK. ^22^Wrightington, Wigan and Leigh NHS Foundation Trust, Wigan, UK. ^23^Nottingham University Hospitals NHS Trust, Nottingham, UK. ^24^ Chelsea and Westminster Hospital NHS Foundation Trust, London, UK. ^25^The Mid Yorkshire Hospitals NHS Trust, Pinderfields, UK. ^26^North Middlesex University Hospital NHS Trust, London, UK. ^27^King's College Hospital NHS Foundation Trust, London, UK. ^28^Guy’s and St Thomas’ NHS Foundation Trust, London, UK. ^29^Yeovil District Hospital NHS Foundation Trust, Yeovil, UK. ^30^East Suffolk and North Essex NHS Foundation Trust, Ipswich, UK. ^31^Northumbria Healthcare NHS Foundation Trust, North Tyneside, UK. ^32^Northampton General Hospital NHS Trust, Northampton, UK. ^33^ Hampshire Hospitals NHS Foundation Trust, Basingstoke, UK. ^34^ North West Anglia NHS Foundation Trust, Peterborough, UK.

**METHODS**

**Study design**

Hospitals were initiated in three phases between 2015 and 2019. On initiation of a site, the hospital started to identify patients on their waiting lists for stress echocardiograms and send them a letter of invitation with a participant information leaflet prior to their appointment. Patients were recruited sequentially on attendance at the stress echo clinic until March 2020. Patients who were referred for stress echocardiography to assess for valve disease, left ventricular outflow tract obstruction or myocardial viability studies were not eligible for recruitment. Hospital size and activity were for each participating centre was collected from national records and number of stress echocardiograms performed by self-report from the participating site.

**RESULTS**

**Patient outcome**

Nine hundred and ninety-two (19.3%) patients tested positive for ischaemia during their stress echocardiogram, of which seven were referred directly for CABG and 378 (38.1%) for elective angiography (371 undergoing invasive coronary angiography and 7 CTCA). Following angiography, 140 (37.0%) patients underwent revascularization, with 105 undergoing PCI and 35 undergoing CABG. A further 67 (17.7%) patients had a severe stenosis (ie. >70% stenosis or positive FFR/iFR) that was managed medically. In the remaining 170 (45.0%) patients, angiography demonstrated no obstructive disease, with one angiogram indeterminate. Eleven of the 615 (1.8%) patients with positive stress echocardiograms who were managed medically and not referred for further investigations presented with an acute coronary syndrome over the following six months. Median time to event was 47 days (IQR 39 – 56 days). Of the 4139 (80.7%) patients who had a negative stress echocardiogram, 95 (2.3%) subsequently underwent angiography, which confirmed non-obstructive disease in 66 (69.5%). In the other 29, 18 subsequently underwent PCI, 2 underwent CABG and 9 had severe disease that was managed medically. Of the 4044 patients who did not have any further investigations, 28 (0.7%) were readmitted with an acute coronary syndrome. Median time to event was 88 days (IQR 34 – 133 days).

Of those undergoing coronary angiography following a positive stress echocardiogram, 114 had single vessel disease of which 52 had disease in the left anterior descending (LAD), 21 in the left circumflex (LCx), 29 in the right coronary artery (RCA)) and 6 patients had disease in other branches. Multi-vessel disease (MVD; defined as the presence of a significant stenosis in at least two epicardial vessels) was present in 93 patients. Of those with MVD, 65 had two-vessel disease (with seven involving the left main stem (LMS)) and 28 patients had three-vessel disease (with 9 cases of LMS involvement).


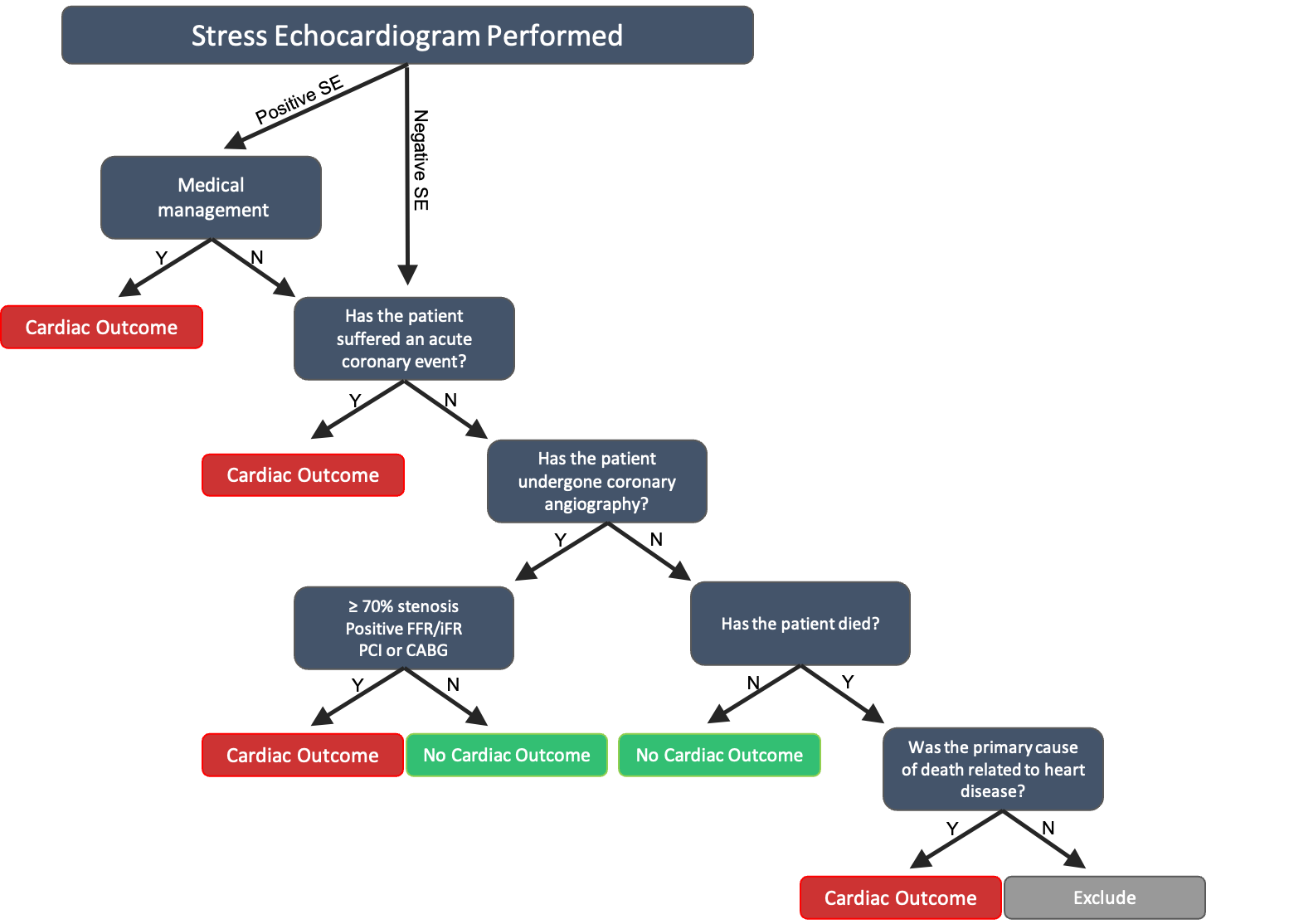


Supplemental Fig. 1: Pathway for outcome adjudication. Data is obtained from a review of the patient’s medical records and via a phone call. This data is then reviewed by a panel of cardiologists to determine patient outcome.


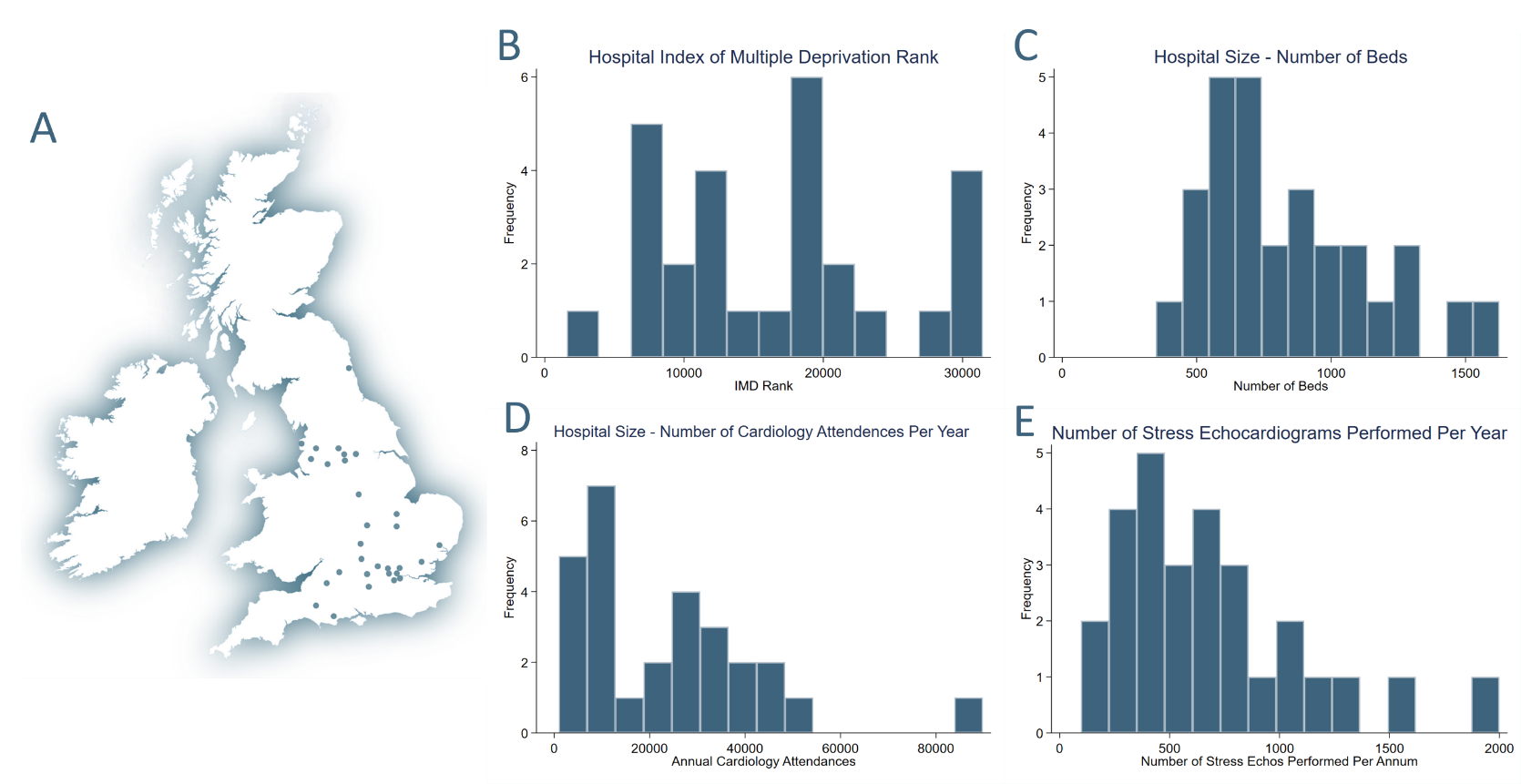


Supplemental Fig. 2: A - Location of the 31 NHS hospitals forming the EVAREST stress echocardiography research network. B – Range of IMD ranks for each hospital, demonstrating the wide range of socio-economic backgrounds represented. C – Hospital size measured by number of beds. D – Hospital cardiology department size measured by number of cardiology attendances per year. E – Volume of stress echocardiograms performed at each hospital per year.

| **Supplemental Table 1: Patient Demographics Separated by Stressor** | | | | |
| --- | --- | --- | --- | --- |
|  | | **Exercise**  **Stress Echo**  **(n=1375)** | **Dobutamine**  **Stress Echo**  **(n=3739)** | **P-valueᵠ** |
| Male (%) | | 831 / 1375 (60.4) | 1981 / 3739 (53.0) | <0.0001 |
| Median Age, Years (IQR) | | 61 (53 – 70) | 68 (59 – 75) | <0.0001 |
| Median BMI, Kg/m^2^ (IQR) | | 27.4 (24.5 – 30.5) | 28.7 (25.3 – 32.3) | <0.0001 |
| Smoking | |  |  |  |
|  | Current Smoker (%) | 142 / 1353 (10.5) | 502 / 3583 (14.0) | <0.001 |
|  | Ex-Smoker (%) | 438 / 1353 (32.4) | 1340 / 3583 (37.4) | <0.001 |
| Hypertension (%) | | 474 / 1258 (37.7) | 1713 / 3672 (46.7) | <0.0001 |
| Hypercholesterolaemia (%) | | 517 / 1258 (41.1) | 1317 / 3672 (35.9) | <0.001 |
| Diabetes Mellitus (%) | | 167 / 1258 (13.3) | 682 / 3672 (18.6) | <0.0001 |
| Family History of Premature CAD (%) | | 23 / 1258 (1.8) | 49 / 3672 (1.3) | 0.208 |
| Peripheral Vascular Disease (%) | | 20 / 1258 (1.6) | 137 / 3672 (3.7) | <0.0001 |
| Pre-existing CAD (%) | | 388 / 1372 (28.3) | 1470 / 3699 (39.7) | <0.0001 |
|  | Previous MI (%) | 169 / 1368 (12.4) | 693 / 3662 (18.9) | <0.0001 |
|  | Previous PCI (%) | 326 / 1371 (23.8) | 1214 / 3668 (33.1) | <0.0001 |
|  | Previous CABG (%) | 67 / 1371 (4.9) | 316 / 3676 (8.6) | <0.0001 |
| Resting RWMA (%) | | 137 / 1372 (10.0) | 570 / 3732 (15.3) | <0.0001 |

Supplemental Table 1: Patient demographics separated by use of exercise stress echocardiography and dobutamine stress echocardiography. Presented as n. / total n. Percentages are quoted in brackets. ᵠP-value for comparison of demographic by type of stress used.

| Supplemental Table 2: Effect of Patient Demographics on Contrast Use | | | |
| --- | --- | --- | --- |
|  | Multivariate Analysis | | |
|  | **Odds Ratio** | **95% CI** | **P-Value** |
| Age | 1.01 | 1.00 – 1.01 | 0.015 |
| Sex (Male) | 0.94 | 0.82 – 1.06 | 0.298 |
| BMI | 1.07 | 1.05 – 1.08 | 0.000 |
| Resting RWMA Present | 1.16 | 0.97 – 1.40 | 0.112 |

Supplemental Table 2: Logistic regression analysis demonstrated that increased age and BMI are significantly associated with an increase in use of LV contrast.


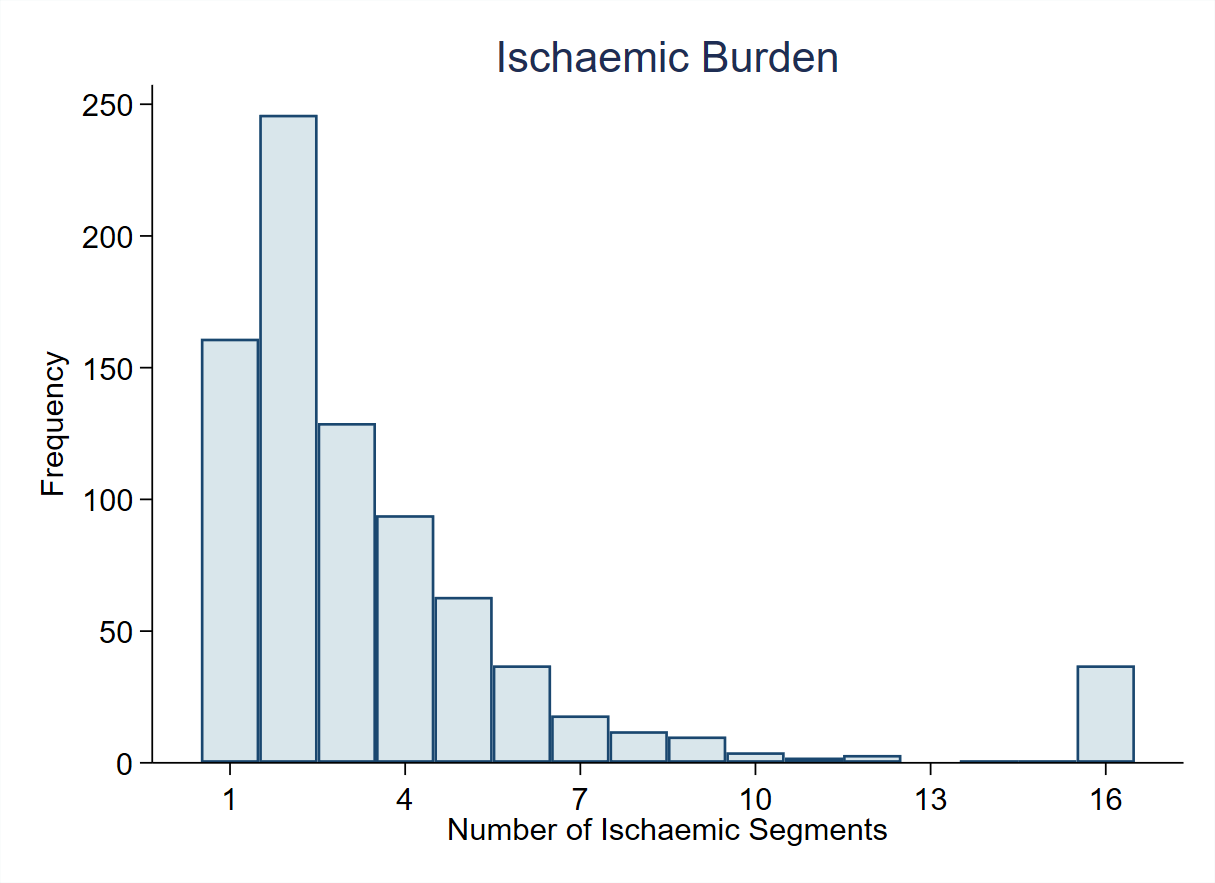


Supplemental Fig. 3: Volume of ischaemia identified during a positive stress echocardiogram, (n=818; NB. burden of ischaemia was not available for 174 patients).
